# The inflammation paradox: Why are Tsimane protected against Western diseases while Westerners are not?

**DOI:** 10.12688/f1000research.14052.2

**Published:** 2018-08-06

**Authors:** Jens Freese, Rainer Johannes Klement, Helmut Lötzerich

**Affiliations:** 1 Institute of Outdoor Sports and Environmental Science, German Sports University Cologne, Cologne, 50933, Germany; 2 Department of Radiotherapy and Radiation Oncology, Leopoldina Hospital Schweinfurt, Schweinfurt, 97422, Germany

**Keywords:** Tsimane, Low-Grade Inflammation, Eifel studies, Non-communicable diseases

## Abstract

We here describe two apparent paradoxes concerning high CRP levels and NCD risk. One has emerged from observational studies in the Amazon region showing that the indigenous Tsimane in Bolivia appear protected against non-communicable diseases (NCDs) such as obesity, type 2 diabetes, and cardiovascular diseases despite increased inflammatory markers. These findings stand in contrast to Western societies, where an increasing body of evidence demonstrates that low-grade-inflammation is the driver of NCDs. The second paradox has emerged from two field studies (Eifel studies) conducted in 2013 and 2014 with Westerners who returned to a simulated Palaeolithic lifestyle in a National park for 4 days. We had detected elevated inflammation markers, despite otherwise anti-inflammatory effects of these interventions as indicated by metabolic blood parameters. We here propose three hypotheses for this second inflammatory paradox.

## Abbreviations

CRP, C-reactive protein; NCD, non-communicable diseases; NK cells, natural killer cells; LGI, low-grade inflammation; LPS, lipopolysaccharides; NFκB, nuclear-factor-kappa-B

## Introduction

Recently, observational studies in the Amazon region showed that the indigenous Tsimane in Bolivia appear protected against non-communicable diseases (NCDs) such as obesity, type 2 diabetes, and cardiovascular diseases, despite increased inflammatory markers ^1^. These findings stand in contrast to Western societies, where an increasing body of evidence demonstrates that low-grade-inflammation (LGI) is the driver of NCDs ^2– 4^.

## Report

Compared to US reference values, Tsimane exhibit markedly high levels of eosinophilic and neutrophilic granulocytes, B lymphocytes and natural killer cells. The leukocyte counts of Tsimans (8,600–12,000 cells/μL) are 1.5 times, and lymphocytes 1.2 to 1.6 times higher than in the US population (6,700–7,900 cells/μL) ^5, 6^. Eosinophilic granulocytes, primarily indicative of parasitic infections, are 7-fold elevated. Consequently, the immunoglobulin E values are also significantly higher (150–200-fold). Important biomarkers for inflammation, such as neutrophil granulocytes (1.2 to 1.6-fold), blood sedimentation (30 mm/h to 15–20 mm/h) and C-reactive protein (CRP) values (higher from infant to adolescence), are also upregulated ^6^. The high CRP levels in Tsimane are in contrast to other indigenous people such as the Hadza hunter-gatherers in Tansania ^7^ or the Shuar hunter-horticulurists in Ecuador ^8^ who show no obvious signs of chronic low-grade inflammation. Thus, chronically high CRP levels in the Tsimane pose an apparent paradox, as high CRP levels due to LGI in Westernized societies correlate with NCD risk. The paradox could be resolved if one assumes that chronic systemic inflammation is a necessary, but not sufficient component in NCD development if certain other factors are simultaneously present. In particular, in the Tsimane the chronic state of infection is thought to stem from a high prevalence of intestinal worms which would i) decrease the absorption of macronutrient in the gut, ii) increase the amount of type 2 anti-inflammatory T helper cells and iii) raise basal metabolic rate, reducing the risk for obesity ^9^. However, the major protective factor against NCD appears to be their lifestyle which prevents the development of typical NCD risk factors such as insulin resistance and adiposity.

In 2013 and 2014, we carried out two field studies (Eifel studies) with Westerners who returned to a simulated Palaeolithic lifestyle in a National park for 4 days ^10, 11^. Contrary to our expectations, in both studies, CRP, the main liver-derived biomarker that displays nonspecific inflammation, had increased by 170% and 67%, respectively. The essential components of these interventions consisted of (i) the conversion to a paleo diet; (ii) the high range of locomotion (15 km/day in the Eifel study 2013, 16.4 km/day in the Eifel study 2014); (iii) a fasting period from 12 to 14 hours per day in conjunction with a low meal frequency resulting in undercaloric energy intake (1567 kcal in the Eifel study 2013, 1747 kcal in the Eifel study 2014). All mentioned factors have been shown to have anti-inflammatory effects ^12– 16^.

## Discussion

There are two interesting observations concerning the indigenous Tsimane in Bolivia and Westeners who returned to a paleolithic lifestyle in the Eifel studies: The former have *chronically* elevated CRP levels despite being practically free of NCDs, while the latter were found to have *acutely* elevated CRP levels despite other physiological changes that would usually be interpreted as protective against NCDs. We provide the following hypothetical explanations for the stimulation of the immune system in the Eifel studies, which are likely to influence one another:

1. Phyto-antibiotics (phytoncides), which plants release into the atmosphere to protect themselves against bacteria and insects, could have stimulated the innate immune system ^17^. As studies from Japan and Korea have shown, so-called "forest bathing" (a multi-day hike through a forest) promotes the formation of high levels of natural killer cells (NK cells). This effect persists for up to 30 days after the intervention ^18, 19^. In addition, forest bathing also increases the activity of the cytolytic proteins perforin, granzyme A and granulysin in NK cells. Walks in the city, on the other hand, do not change the NK cell population or its activity ^19^. These effects could have contributed to the increase in CRP levels in the Eifel studies, as most of the time participants spent in a forest area.
2. The radical change from a near-sterile to a natural environment may have prompted the innate immune system to anticipate and prophylactically protect the organism against pathogens such as bacteria, parasites, fungi, and other microorganisms. Danger signals, called exogenous pathogen associated molecular patterns and endogenous danger associated molecular patterns, activate the innate immune system via Toll-like receptors, which can trigger a rapid antibacterial inflammatory response. This mechanism of action may have led to the development of an acute phase response. In contrast to LGI, substances such as lipoxins, resolvins and protectins are formed in acute inflammation in order to end the inflammatory process ^20, 21^. Since no follow-up measurements were made in the Eifel studies, this hypothesis is currently only speculative.
3. Despite the fact that the participants in the Eifel studies were in good mental and physical health, the level of physical stress due to the high workload combined with calorie restriction conditions could have induced a moderate acute phase response. Indeed, acute and transient elevations of CRP levels, inflammatory cytokines (in particular IL-6) and shifts in leucocyte patterns typically occur after strenuous exercise ^22– 24^. The latter include elevations in neutrophils and decreases in eopsinophils and lymphocytes, changes that have also been observed in our Eifel study participants ^10, 11^. Therefore, this hypothesis appears to be the best confirmed by the data. The acute increase in CRP levels could thereby be caused by several mechanisms including increased catecholamine and cortisol levels ^23^ or endotoxemia through bacterial lipopolysaccharides (LPS) caused by increased cell destruction and gut leakage ^25^. LPS activate the innate immune system via Toll-like receptors and stimulate the activation of nuclear-factor-kappa-B (NFκB) intracellularly, leading to pro-inflammatory cytokine secretion ^26^. A by-product of cell destruction is uric acid, which stimulates the release of CRP in the liver as part of the acute immune response ^27, 28^. In turn, CRP stimulates the production of antibodies from B lymphocytes to kill pathogens ^29^. Due to the high range of locomotion in both Eifel studies, uric acid might have played a prominent role in stimulating the immune system. Since uric acid has not been measured, future studies should include this marker to provide a possible confirmation of this hypothesis.

## Outlook

The fact that a chronic inflammatory situation in Tsimans persists despite practical non-existence of NCDs, while it is associated with NCDs in Westerners, appears paradoxical. A second apparent paradox concerns the acutely elevated CRP levels in Westeners returning to a Paleolithic lifestyle despite otherwise beneficial metabolic effects usually associated with a decrease in LGI. We have discussed three different hypotheses to solve this second paradox. While the “forest-bathing” and “danger anticipation” hypotheses are currently only speculative due to the lack of relevant measurements, a mild-to-moderate acute-phase response due to the strenuous physical activity is consistent with the available data. However, the data cannot clearly constitute evidence for one of these hypotheses against any other, in part because their likelihood under the “forest-bathing” and “danger anticipation” hypotheses remains to be determined. Finally it remains to be examined if, when and how the acute elevations in CRP levels would resolve if the participants would remain in Paleolithic living conditions. Interestingly, a study similar to the Eifel studies involving a 10-day trip through the Pyrenees also reported a significant mild increase in CRP levels after the trip, showing that the acute phase repsonse could be sustained at least up to 10 days ^30, 31^.

## Data availability

The data referenced by this article are under copyright with the following copyright statement: Copyright: © 2018 Freese J et al.

Data associated with the article are available under the terms of the Creative Commons Zero "No rights reserved" data waiver (CC0 1.0 Public domain dedication). 

No data are associated with this article.
