# Supplementary material for: The inflammation paradox: Why are Tsimane protected against Western diseases while Westerners are not?
Source: F1000Res. 2018 Aug 6;7:252. Originally published 2018 Mar 1. [Version 2] doi: 10.12688/f1000research.14052.2 (PMC6685450; doi:10.12688/f1000research.14052.2)
Supplement: Peer review history

# Peer review: Reviewer response for version 2

**DOI:** 10.5256/f1000research.17155.r50908

**Published:** 2019-08-06

**Authors:** J. Josh Snodgrass

**Affiliations:** 1 Department of Anthropology, University of Oregon, Eugene, OR, USA

I have the luxury of reading this article after revisions based on two thorough reviews, both of which raise important points and make excellent suggestions for improvement. The new version of the article has been substantively revised in response to these reviews. The present version of the article, while highly speculative, does raise some interesting and important issues and starts a conversation that will generate additional testing and data collection.

 Several points are worth discussing in more detail. I appreciate that the authors are much more clear in the revised article that chronic inflammation in the Tsimane is not protective against NCDs and instead that it is a result of the absence of certain NCD risk factors that are common in other populations. My research group has published Shuar CRP data cited in the article as well as comparative immune studies of Tsimane and Shuar. A key point that’s mentioned in the present article yet not elaborated on here or really elsewhere is the divergent CRP values in Shuar and Tsimane despite both being subsistence-based populations in the Amazon. It seems likely that differences in disease ecology between the two groups, especially related to helminths, influences inflammatory profile and other immune differences. Although baseline CRP is low among Shuar, they do experience acute CRP elevations in response to various challenges. It would be useful to look at helminth species (extent of helminth coinfection) and intensity in each of the populations coupled with immune work to see the extent to which chronically elevated inflammation is reflective of repeated or constant helminth infections. Clearly more work needs to be done to identify underlying contributors to inflammation in these types of populations, and their contribution to disease risk. I appreciate that this article drives that conversation forward.

 The Eifel Studies, while interesting and potentially useful for unraveling contributions to inflammation, are very brief in duration and with very small sample sizes. The studies need to be of longer duration to fully understand the time-course of the various biomarkers (it’s impossible to interpret changes in a small sample in only 4 days), as well as need to include measurement of more variables of interest. I think I would feel more comfortable if the authors made it more clear that these studies are very preliminary. And of course then make plans for additional research!

 As for the proposed explanations, while highly speculative, they do advance the conversation and potentially lead to testable hypotheses that can be examined using new data collected in studies like those of Eifel as well as in populations like the Tsimane and Shuar.

I confirm that I have read this submission and believe that I have an appropriate level of expertise to confirm that it is of an acceptable scientific standard.

# Peer review: Reviewer response for version 2

**DOI:** 10.5256/f1000research.17155.r36833

**Published:** 2018-10-23

**Authors:** Michael D. Gurven

**Affiliations:** 1 Department of Anthropology, University of California, Santa Barbara, Santa Barbara, CA, USA

I confirm that I have read this submission and believe that I have an appropriate level of expertise to confirm that it is of an acceptable scientific standard.

# Peer review: Reviewer response for version 1

**DOI:** 10.5256/f1000research.15283.r31345

**Published:** 2018-04-06

**Authors:** Colette Berbesque, Baptiste Sadoughi

**Affiliations:** 1 Centre for Research in Evolutionary, Social and Inter-Disciplinary Anthropology, University of Roehampton, London, UK

The authors statement is organised around two discoveries: the highly inflammatory immune profile in Tsimane associated with low prevalence of NCDs, and the increased inflammation in westerners whom participated in a 'Paleo-trek' ^1^. From these observations, FKL reach the conclusion that chronic inflammation in the Tsimane protects against NCDs and propose explanations for why this might be the case. We will discuss this assumption in the following sections.

 First, we disagree with the assumption that Tsimane people stand as an adequate model for the study of the relationship between Low-grade inflammation (LGI) and non-communicable diseases (NCDs). LGI, associated with coronary diseases and other NCDs in westerners, is usually not triggered by specific stimuli such as infection or injury ^2^, but strongly associated with other risk factors such as stressful situations ^3^ and excess of adipose tissue ^4^. Tismanes are less mobile than most hunter-gatherers, such as the Hadza - who demonstrate low CPR ^5^. This increased sedentisation may be part of the reason they face a heavy parasitic load and bacterial infections ^6^ likely responsible for their hightened inflammatory response ^7^, along with low levels of cholesterol, LDL and HDL. It is also noteworthy that the major comparative study on Tsimane immune function failed to show that after childhood there was any significant increase in CRP, a major low-grade inflammatory marker ^7^. The hypothesis that the parasitic burden triggering Th2 response may enhance anti-inflammatory cytokine levels and thus protect against NCDs is interesting. However, the absence of major NCDs risk factors, such as chronic stress, insulin-resistance and elevated fatty acid levels, brings into question the underlying assumption that the Tsimane may face common NCDs. 

 Finally, we strongly reject the authors' conclusion that inflammation may protect against NCDs in Tsimane. Indeed, CRP levels were found to be a positive predictor of non-null coronary artery calcium scores ^8^, suggesting CRP might actually be predictive of atherosclerosis, a major NCD, in Tsimane as well.

 According to the authors, the inflammatory state of the Tsimane could be partly explained by environmental factors also responsible for the pro-inflammatory profile observed in the participants of the Eifel experiment. We will review and discuss two of the hypotheses proposed by FKL to explain this result.

 First, a ‘forest bathing’ effect would have enhanced innate immune response and therefore result in an increase in CRP levels. Although we do not wish to undermine the potential role of the ‘forest bathing’ effect, to the best of our knowledge no evidence so far suggests that it could have an impact on CRP levels. Most studies focus on NK cells, cortisol levels and bio-markers of endothelial function. As CRP elevation in the Eifel participants failed to reach significance and NK cells were not measured, the ‘forest bathing’ effect remains speculative.

 Second, as suggested by the authors, exposure to exogenous pathogen associated-molecules could have caused a shift towards Th1 response in Eifel participants. However, there is no evidence to support the assumption that this shift was 'prophylactic' as participants may have well indeed face acute infection from small wounds or respiratory pathogens in a forest environment. In order to strengthen their theoretical argument, the authors could provide references regarding the resolution of chronic inflammation following an episode of acute inflammation.

 Finally, we would like to suggest another potential origin for the pro-inflammatory immune profile in the Eifel participants. It seems participants exhibit the typical leukocyte count induced by stress and exercise ^9^: neutrophilia, lymphopenia and eosinopenia. Such leukocyte pattern is supposedly the result of cortisol-induced cell-migration into peripheral tissue ^10^ and does not fit with Tsimane leukocyte pattern ^7^. Furthermore, exercise-induced response has been shown to gradually decrease in trained athletes. Long-term training is eventually followed by the release of anti-inflammatory cytokines ^11^^,^^12^. Such difference between initial-shock immune response to intense exercise, as undertaken by untrained Eifel participants, and long-term immune adaptation to physical activity brings into question the reliability of the leukogram patterns measured on the fourth day of trek in the Eifel study. A shift towards a more anti-inflammatory pattern could therefore be expected in the Eifel participants after acclimatisation to this new lifestyle, regardless of environmental factors. It is therefore highly speculative that the Eifel participants’ immune profile would be a good model for the immune pattern of a Paleo-population.

We confirm that we have read this submission and believe that we have an appropriate level of expertise to confirm that it is of an acceptable scientific standard, however we have significant reservations, as outlined above.

## References

[1] : Back to the Future. Metabolic Effects of a 4-Day Outdoor Trip Under Simulated Paleolithic Conditions – New Insights from The Eifel Study. *Journal of Evolution and Health*.2016;1(1) : 10.15310/2334-3591.1035 10.15310/2334-3591.1035

[2] : Stimulation of systemic low-grade inflammation by psychosocial stress. *Psychosom Med*.2014;76(3) : 10.1097/PSY.0000000000000049 181-9 10.1097/PSY.0000000000000049 24608036

[3] : Inflammation as a psychophysiological biomarker in chronic psychosocial stress. *Neurosci Biobehav Rev*.2010;35(1) : 10.1016/j.neubiorev.2009.12.012 115-21 10.1016/j.neubiorev.2009.12.012 20026349

[4] : The relation of body fat mass and distribution to markers of chronic inflammation. *Int J Obes Relat Metab Disord*.2001;25(10) : 10.1038/sj.ijo.0801792 1407-15 10.1038/sj.ijo.0801792 11673759

[5] : Physical activity patterns and biomarkers of cardiovascular disease risk in hunter-gatherers. *Am J Hum Biol*.2017;29(2) : 10.1002/ajhb.22919 10.1002/ajhb.22919 27723159

[6] : Reproductive trade-offs in extant hunter-gatherers suggest adaptive mechanism for the Neolithic expansion. *Proc Natl Acad Sci U S A*.2016;113(17) : 10.1073/pnas.1524031113 4694-9 10.1073/pnas.1524031113 27071109PMC4855554

[7] : Immune function in Amazonian horticulturalists. *Ann Hum Biol*.2016;43(4) : 10.1080/03014460.2016.1189963 382-96 10.1080/03014460.2016.1189963 27174705PMC4976077

[8] : Coronary atherosclerosis in indigenous South American Tsimane: a cross-sectional cohort study. *The Lancet*.2017;389(10080) : 10.1016/S0140-6736(17)30752-3 1730-1739 10.1016/S0140-6736(17)30752-3 PMC602877328320601

[9] : Circulating cytokines and hormones with immunosuppressive but neutrophil-priming potentials rise after endurance exercise in humans. *Eur J Appl Physiol*.2000;81(4) : 10.1007/s004210050044 281-7 10.1007/s004210050044 10664086

[10] : The use of leukocyte profiles to measure stress in vertebrates: a review for ecologists. *Functional Ecology*.2008;22(5) : 10.1111/j.1365-2435.2008.01467.x 760-772 10.1111/j.1365-2435.2008.01467.x

[11] : Endurance exercise causes interaction among stress hormones, cytokines, neutrophil dynamics, and muscle damage. *J Appl Physiol (1985)*.1999;87(4) : 10.1152/jappl.1999.87.4.1360 1360-7 10.1152/jappl.1999.87.4.1360 10517764

[12] : Physical exercise modulates the homeostasis of human regulatory T cells. *J Allergy Clin Immunol*.2016;137(5) : 10.1016/j.jaci.2015.10.035 1607-1610.e8 10.1016/j.jaci.2015.10.035 26774657

# Response

**Published:** 2018-07-26

**Authors:** Jens Freese

**Affiliations:** Deutsche Sporthochschule Köln, Germany

The authors statement is organised around two discoveries: the highly inflammatory immune profile in Tsimane associated with low prevalence of NCDs, and the increased inflammation in westerners whom participated in a 'Paleo-trek' ^1^. From these observations, FKL reach the conclusion that chronic inflammation in the Tsimane protects against NCDs and propose explanations for why this might be the case. We will discuss this assumption in the following sections.

 First, we disagree with the assumption that Tsimane people stand as an adequate model for the study of the relationship between Low-grade inflammation (LGI) and non-communicable diseases (NCDs). LGI, associated with coronary diseases and other NCDs in westerners, is usually not triggered by specific stimuli such as infection or injury ^2^, but strongly associated with other risk factors such as stressful situations ^3^ and excess of adipose tissue ^4^. Tismanes are less mobile than most hunter-gatherers, such as the Hadza - who demonstrate low CPR ^5^. This increased sedentisation may be part of the reason they face a heavy parasitic load and bacterial infections ^6^ likely responsible for their hightened inflammatory response ^7^, along with low levels of cholesterol, LDL and HDL. It is also noteworthy that the major comparative study on Tsimane immune function failed to show that after childhood there was any significant increase in CRP, a major low-grade inflammatory marker ^7^. The hypothesis that the parasitic burden triggering Th2 response may enhance anti-inflammatory cytokine levels and thus protect against NCDs is interesting. However, the absence of major NCDs risk factors, such as chronic stress, insulin-resistance and elevated fatty acid levels, brings into question the underlying assumption that the Tsimane may face common NCDs. 

**Answer: We apologize if we seemed to have suggested the Tsimane as an adequate model for studying the connection between LGI and NCD. This was not our aim. Rather, our aim was to point out the Tsimane as an interesting case of indigenous people who are apparently free from the NCD that plague the Western societies despite having chronically elevated CRP levels. This should make clear that they are not an adequate model, but rather an exceptional case worth further studying. In our revised manuscript, we strengthen the notion that Tsimane do not face the usual risk factors for NCDs that Westeners do, agreeing with your argument above. This is probably the best explanantion for their low incidence of NCDs.**

 Finally, we strongly reject the authors' conclusion that inflammation may protect against NCDs in Tsimane. Indeed, CRP levels were found to be a positive predictor of non-null coronary artery calcium scores ^8^, suggesting CRP might actually be predictive of atherosclerosis, a major NCD, in Tsimane as well.

**Answer: We are sorry for making causal claims where the data do not clearly support any. We have been more careful with our formulations in the revised manuscript, refraining from making any causal claims.**

 According to the authors, the inflammatory state of the Tsimane could be partly explained by environmental factors also responsible for the pro-inflammatory profile observed in the participants of the Eifel experiment. We will review and discuss two of the hypotheses proposed by FKL to explain this result.

 First, a ‘forest bathing’ effect would have enhanced innate immune response and therefore result in an increase in CRP levels. Although we do not wish to undermine the potential role of the ‘forest bathing’ effect, to the best of our knowledge no evidence so far suggests that it could have an impact on CRP levels. Most studies focus on NK cells, cortisol levels and bio-markers of endothelial function. As CRP elevation in the Eifel participants failed to reach significance and NK cells were not measured, the ‘forest bathing’ effect remains speculative.

**Answer: We agree that this hypothesis is currently only speculative until further measurements have been made which are able to distinguish it from other hypotheses such as the acute phase response due to strenuous physical activity. We have re-phrased the Outlook section accordingly. The same is true for our second hypothesis. Nevertheless, we consider them possible explanations that should be considered in data interpretation. Our future research is planned to more specifically address the forest bathing hypothesis.**

 Second, as suggested by the authors, exposure to exogenous pathogen associated-molecules could have caused a shift towards Th1 response in Eifel participants. However, there is no evidence to support the assumption that this shift was 'prophylactic' as participants may have well indeed face acute infection from small wounds or respiratory pathogens in a forest environment. In order to strengthen their theoretical argument, the authors could provide references regarding the resolution of chronic inflammation following an episode of acute inflammation.

**Answer: We had already noted that this hypothesis is currently only speculative. However, since we are mainly interested in explaining the acute rise of CRP levels, we have deleted any hypothesizing that this acute phase response could have led to the resolution of chronic LGI. An example for the resolution of chronic inflammation after acute stimulation of the immune system (although not neccessarily translatable to all NCDs) is the spontaneous remission of cancer after an acute episode of fever that is now more or less officially acknowledged (e.g. Jessy T (2011): Immunity over inability: The spontaneous regression of cancer. J Nat Sci Biol Med; 2(1): 43–49). Other examples from medical practice include treatment of chronic** **tendonitis through re-injury or using low-dose ionizing radiation to treat chronic inflammatory conditions.**

 Finally, we would like to suggest another potential origin for the pro-inflammatory immune profile in the Eifel participants. It seems participants exhibit the typical leukocyte count induced by stress and exercise ^9^: neutrophilia, lymphopenia and eosinopenia. Such leukocyte pattern is supposedly the result of cortisol-induced cell-migration into peripheral tissue ^10^ and does not fit with Tsimane leukocyte pattern ^7^. Furthermore, exercise-induced response has been shown to gradually decrease in trained athletes. Long-term training is eventually followed by the release of anti-inflammatory cytokines ^11^ ^,^ ^12^. Such difference between initial-shock immune response to intense exercise, as undertaken by untrained Eifel participants, and long-term immune adaptation to physical activity brings into question the reliability of the leukogram patterns measured on the fourth day of trek in the Eifel study. A shift towards a more anti-inflammatory pattern could therefore be expected in the Eifel participants after acclimatisation to this new lifestyle, regardless of environmental factors. It is therefore highly speculative that the Eifel participants’ immune profile would be a good model for the immune pattern of a Paleo-population.

**Answer: Thank you for these thoughts which go along the lines of our third hypothesis, namely that the sudden physical activity increase would be responsible for the acute CRP increase. We have included this additional mechanism in our discussion and re-wrote a large part of the third hypothesis. We think that the revision now also distinguishes more clearly between the chronic high CRP levels of the Tsimane and the acute elevations observed in the Eifel studies. In no way did we intent to propose that the immune profile of the latter is a good model for the former.**

# Peer review: Reviewer response for version 1

**DOI:** 10.5256/f1000research.15283.r31343

**Published:** 2018-03-12

**Authors:** Michael D. Gurven, Caleb Finch

**Affiliations:** 1 Department of Anthropology, University of California, Santa Barbara, Santa Barbara, CA, USA; 2 Leonard Davis School of Gerontology & Dornsife College, University of Southern California, Los Angeles, CA, USA

We appreciate that Freese and colleagues [FKL] attempt to understand what they refer to as the “inflammation paradox”, i.e. that people may experience high chronic levels of circulating inflammation (as measured by biomarkers such as high sensitivity C-reactive protein (CRP)), yet not succumb to the diseases such as heart disease and diabetes usually attributed to such high exposure in urban settings. We first documented this pattern among Tsimane Amerindians in Bolivia in 2009 when we found no cases of peripheral arterial disease among older adults despite high average levels of CRP, and no relationship between CRP and the continuous ankle-brachial index used to assess peripheral arterial disease risk ^1^. More recently, we reported minimal coronary artery calcification that progressed 25 years more slowly than in the US,, and again, without any relationship with CRP ^2^.

 FKL assert that the typically high inflammation the Tsimane experience may be protective against non-communicable diseases (NCDs), and that their healthy hearts may not be due simply to their having protective diets, high moderate-to-vigorous activity levels and other lifestyle factors. We interpret their position that high inflammation is protective based on their discussion of field Eifel studies which showed CRP elevations after an attempt to mimic a more traditional “Paleo” lifestyle on a 4 day forest trek with low-carb diet, caloric restriction and semi-fasting. They propose three reasons for why CRP might be elevated under these (and presumably Tsimane conditions), but yet have no relationship with NCDs and their inflammation-related risk factors like smoking and obesity: 1) forest bathing may induce innate immune activity from plants releasing phyto-antibiotics into the atmosphere; 2) trekking in the “natural environment” (but not the city) may induce anticipatory immune defenses; and 3) caloric restriction combined with vigorous activity may have led to “cell depletion” and increased innate immune activity, including CRP, via uric acid byproducts.

 First, we disagree with their conclusion that a “chronic inflammatory situation in Tsimans [sic]  protects against NCD”. This has never been shown. Rather, we have just shown that Tsimane do not appear to suffer from NCDs like heart disease and diabetes, despite  their  elevated systemic inflammation. Elsewhere, we have proposed that high inflammation may not lead to increased heart disease risk under certain conditions that may be particular for traditional subsistence populations like the Tsimane ^3^^,^^4^. High systemic inflammation might not lead to greater NCD risk when combined with: 1) low LDL, a physically demanding lifestyle and minimal obesity; 2) chronic helminthic infections that: a) modulate immune function toward more anti-inflammatory Th2 activity that helps prevents systemic inflammation from damaging arteries, b) increase basal metabolic rate and lower obesity risk; c) reduce blood lipids such as LDL cholesterol and triglycerides, and blood glucose. Also notable is their limited exposure to tobacco smoke.

 Second, while the Eifel study results suggest that adopting a more “Paleo”-like lifestyle can improve cardiometabolic biomarkers, we disagree that they show that higher inflammation (or innate immune activity in general) typifies hunter-gatherers or other subsistence populations. While CRP increased by 67% after the four day forest trek of healthy adults, this increase was not statistically significant (see Table 4). Thus, given the small sample size (n=25), these speculations have limited basis and are premature. Moreover, we see other issues with generalizing from the Eifel study. Half of the study participants did not engage in regular physical activity (52% <3 hrs/week). Heavy physical activity could lead to acute increases in inflammation, whereas long-term effects of regular exercise are usually associated with reduced chronic low grade inflammation. It is also possible that during the experiment, some participants incurred minor injuries or acute infections, both of which could result in higher CRP. Psychosocial stress, as might occur under the Eifel study conditions of food restriction for example, can also increase inflammation, especially IL-6, IL-1B and CRP ^5^.

 While Tsimane show evidence of chronic, elevated CRP across much of the life course ^6^^,^^7^, it is yet to be determined how representative this pattern is among small-scale subsistence populations. Shuar forager-horticulturalists of Ecuador show elevated CRP only when infected, but otherwise have low baseline CRP levels ^8^. A small sample of Hadza hunter-gatherers also showed reasonably low CRP levels (74% of 23 adults had CRP < 3 mg/L) ^9^.

 Anticipatory stimulation of the innate immune system in the forest is an interesting idea; even visual exposure to photographs with disease cues (e.g. sneezing) can increase IL-6 by 24% compared to controls shown neutral stimuli ^10^. However, while short-term changes in other biomarkers like blood lipids and blood glucose may be generalizable from the Eifel study to a long-term study, it is unclear whether any acute innate immune activity observed over a 4 day period would sustain in a longer-term study after habituation. As FKL argue, there are many other reasons CRP can be elevated that have little to do with inflammation, but might still be related to tissue stress or injury ^11^.

We confirm that we have read this submission and believe that we have an appropriate level of expertise to confirm that it is of an acceptable scientific standard, however we have significant reservations, as outlined above.

## References

[1] : Inflammation and infection do not promote arterial aging and cardiovascular disease risk factors among lean horticulturalists. *PLoS One*.2009;4(8) : 10.1371/journal.pone.0006590 e6590 10.1371/journal.pone.0006590 19668697PMC2722089

[2] : Coronary atherosclerosis in indigenous South American Tsimane: a cross-sectional cohort study. *The Lancet*.2017;389(10080) : 10.1016/S0140-6736(17)30752-3 1730-1739 10.1016/S0140-6736(17)30752-3 PMC602877328320601

[3] : Cardiovascular disease and type 2 diabetes in evolutionary perspective: a critical role for helminths?. *Evol Med Public Health*.2016; 10.1093/emph/eow028 10.1093/emph/eow028 27666719PMC5101910

[4] : Blood lipids, infection, and inflammatory markers in the Tsimane of Bolivia. *Am J Hum Biol*.22(6) : 10.1002/ajhb.21074 731-40 10.1002/ajhb.21074 20721985PMC3537506

[5] : The effects of acute psychological stress on circulating inflammatory factors in humans: a review and meta-analysis. *Brain Behav Immun*.2007;21(7) : 10.1016/j.bbi.2007.03.011 901-12 10.1016/j.bbi.2007.03.011 17475444

[6] : Aging and inflammation in two epidemiological worlds. *J Gerontol A Biol Sci Med Sci*.2008;63(2) :196-9 1831445710.1093/gerona/63.2.196PMC2952348

[7] : Immune function in Amazonian horticulturalists. *Ann Hum Biol*.2016;43(4) : 10.1080/03014460.2016.1189963 382-96 10.1080/03014460.2016.1189963 27174705PMC4976077

[8] : Analysis of variability of high sensitivity C-reactive protein in lowland Ecuador reveals no evidence of chronic low-grade inflammation. *Am J Hum Biol*.24(5) : 10.1002/ajhb.22296 675-81 10.1002/ajhb.22296 22639072

[9] : Physical activity patterns and biomarkers of cardiovascular disease risk in hunter-gatherers. *Am J Hum Biol*.2017;29(2) : 10.1002/ajhb.22919 10.1002/ajhb.22919 27723159

[10] : Mere visual perception of other people's disease symptoms facilitates a more aggressive immune response. *Psychol Sci*.2010;21(5) : 10.1177/0956797610368064 649-52 10.1177/0956797610368064 20483842

[11] : What does minor elevation of C-reactive protein signify?. *Am J Med*.2006;119(2) : 10.1016/j.amjmed.2005.06.057 166.e17-28 10.1016/j.amjmed.2005.06.057 16443421

# Response

**Published:** 2018-07-26

**Authors:** Jens Freese

**Affiliations:** Deutsche Sporthochschule Köln, Germany

We appreciate that Freese and colleagues [FKL] attempt to understand what they refer to as the “inflammation paradox”, i.e. that people may experience high chronic levels of circulating inflammation (as measured by biomarkers such as high sensitivity C-reactive protein (CRP)), yet not succumb to the diseases such as heart disease and diabetes usually attributed to such high exposure in urban settings. We first documented this pattern among Tsimane Amerindians in Bolivia in 2009 when we found no cases of peripheral arterial disease among older adults despite high average levels of CRP, and no relationship between CRP and the continuous ankle-brachial index used to assess peripheral arterial disease risk ^1^. More recently, we reported minimal coronary artery calcification that progressed 25 years more slowly than in the US,, and again, without any relationship with CRP ^2^.

 FKL assert that the typically high inflammation the Tsimane experience may be protective against non-communicable diseases (NCDs), and that their healthy hearts may not be due simply to their having protective diets, high moderate-to-vigorous activity levels and other lifestyle factors. We interpret their position that high inflammation is protective based on their discussion of field Eifel studies which showed CRP elevations after an attempt to mimic a more traditional “Paleo” lifestyle on a 4 day forest trek with low-carb diet, caloric restriction and semi-fasting. They propose three reasons for why CRP might be elevated under these (and presumably Tsimane conditions), but yet have no relationship with NCDs and their inflammation-related risk factors like smoking and obesity: 1) forest bathing may induce innate immune activity from plants releasing phyto-antibiotics into the atmosphere; 2) trekking in the “natural environment” (but not the city) may induce anticipatory immune defenses; and 3) caloric restriction combined with vigorous activity may have led to “cell depletion” and increased innate immune activity, including CRP, via uric acid byproducts.

 First, we disagree with their conclusion that a “chronic inflammatory situation in Tsimans [sic]  protects against NCD”. This has never been shown. Rather, we have just shown that Tsimane do not appear to suffer from NCDs like heart disease and diabetes, despite  their  elevated systemic inflammation. Elsewhere, we have proposed that high inflammation may not lead to increased heart disease risk under certain conditions that may be particular for traditional subsistence populations like the Tsimane ^3^ ^,^ ^4^. High systemic inflammation might not lead to greater NCD risk when combined with: 1) low LDL, a physically demanding lifestyle and minimal obesity; 2) chronic helminthic infections that: a) modulate immune function toward more anti-inflammatory Th2 activity that helps prevents systemic inflammation from damaging arteries, b) increase basal metabolic rate and lower obesity risk; c) reduce blood lipids such as LDL cholesterol and triglycerides, and blood glucose. Also notable is their limited exposure to tobacco smoke.

**Answer: We agree that there are no data showing that chronic inflammation in Tsimans protects them against NCD (correlation is not causation). We have erased this claim. Rather, we agree that the best explantion is probably the absence of typical NCD risk factors. Therefore, the Tsimane are noteworthy for being an example that chronic high inflammation is not sufficient for increasing NCD risk, given that other conditions are satisfied as you mentioned. This is now emphasized in the revision.**

 Second, while the Eifel study results suggest that adopting a more “Paleo”-like lifestyle can improve cardiometabolic biomarkers, we disagree that they show that higher inflammation (or innate immune activity in general) typifies hunter-gatherers or other subsistence populations. While CRP increased by 67% after the four day forest trek of healthy adults, this increase was not statistically significant (see Table 4). Thus, given the small sample size (n=25), these speculations have limited basis and are premature. Moreover, we see other issues with generalizing from the Eifel study. Half of the study participants did not engage in regular physical activity (52% <3 hrs/week). Heavy physical activity could lead to acute increases in inflammation, whereas long-term effects of regular exercise are usually associated with reduced chronic low grade inflammation. It is also possible that during the experiment, some participants incurred minor injuries or acute infections, both of which could result in higher CRP. Psychosocial stress, as might occur under the Eifel study conditions of food restriction for example, can also increase inflammation, especially IL-6, IL-1B and CRP ^5^.

**Answer**: **You are correct that high-grade inflammation is not a general hallmark of hunter-gatherer populations, and we apologize if we seem to have made such a statement. Our goal was simply to propose several possible hypotheses fort he observed acute elevations in CRP in the Eifel studies, partly in preparation for a new study that is going to start this summer. We also state in the revision that the data are not able to provide evidence for one of our hypotheses over any other, so that future studies should particularly put these hypotheses to test.**

 While Tsimane show evidence of chronic, elevated CRP across much of the life course ^6^ ^,^ ^7^, it is yet to be determined how representative this pattern is among small-scale subsistence populations. Shuar forager-horticulturalists of Ecuador show elevated CRP only when infected, but otherwise have low baseline CRP levels ^8^. A small sample of Hadza hunter-gatherers also showed reasonably low CRP levels (74% of 23 adults had CRP < 3 mg/L) ^9^.

**Answer:** **Thank you for pointing this out. We added the references to these studies that indicate that the Tsimane CRP levels are apparently higher than that of other indegenous people.**

 Anticipatory stimulation of the innate immune system in the forest is an interesting idea; even visual exposure to photographs with disease cues (e.g. sneezing) can increase IL-6 by 24% compared to controls shown neutral stimuli ^10^. However, while short-term changes in other biomarkers like blood lipids and blood glucose may be generalizable from the Eifel study to a long-term study, it is unclear whether any acute innate immune activity observed over a 4 day period would sustain in a longer-term study after habituation. As FKL argue, there are many other reasons CRP can be elevated that have little to do with inflammation, but might still be related to tissue stress or injury ^11^.

**Answer: We agree that it is unclear if and when the elevated CRP levels found in the Eifel study participants would have decreased again. However, a similar study has found increased CRP levels after a 10-day trip through the wilderness. This is stated now in the last two sentences of the conclusions section: “In particular it remains to be determined, if, when and how the acute elevations in CRP level resolve if the participants would remain in Paleolithic living conditions. Interestingly, a study similar to the Eifel studies involving a10-day trip through the Pyrenees also reported a significant mild increase in CRP levels after the trip, showing that the acute phase repsonse could be sustained at least up to 10 days (29-30).“**
